# Bilateral femoral fractures during birth, in a neonate with malformation complex with stiffness

**DOI:** 10.1002/ccr3.2877

**Published:** 2020-04-27

**Authors:** Nikolaos Laliotis, Konstantia Tsoni, Panagiotis Konstantinidis, Eleni Agakidou, Chrysanthos Chrysanthou

**Affiliations:** ^1^ Orthopaedic Department Interbalkan Medical Center Thessaloniki Greece; ^2^ 1st Department of Neonatology and NICU Aristotle University of Thessaloniki Ippokration General Hospital Thessaloniki Greece

**Keywords:** femoral fractures, malformation complex, multiple congenital contractures, neonatal

## Abstract

Fetuses with prenatal diagnosis of reduced mobility and malformation are at increased risk to sustain neonatal femoral fractures. Labor difficulties even after an elective cesarean section may trigger the fractures of the long bones.

## Graphical abstract

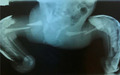

## 1 INTRODUCTION

We describe a neonate, with malformation complex, that sustained bilateral femoral fractures. Severe stiffness and peculiar shape of the body triggered the injury. Treatment with Gallow's traction was applied, and the fractures healed within 3 weeks. We plan further treatment of the deformities of the limbs and spine.

Neonatal femoral fractures are rare, and only sporadic case reports have presented perinatal fractures of the femur. Breech presentation, vaginal delivery, and cesarean section (CS) of low or heavy weight babies have been reported as risk factors of perinatal femoral fractures. The most common symptoms are swelling of the leg, absence of movement, and discomfort while changing nappies.^1^, ^2^ Bilateral femoral fractures are extremely rare. They may be expected in babies with underlying disease such as severe form of osteogenesis imperfecta or arthrogryposis.^3^, ^4^, ^5^, ^6^

The aim of this study was to present a rare case of a neonate with malformation complex with severe contractures and bilateral femoral fractures.

## 2 CASE REPORT

A female neonate was born from a second parous mother, at 40 weeks of gestation, with birth weight of 2230 g. The parents and their first child were normal. Prenatal ultrasound (US) examination showed reduced fetal movements, unrecognizable sex because of difficult visualization of the genitourinary area, and equinovarus feet.

**Figure 1:**
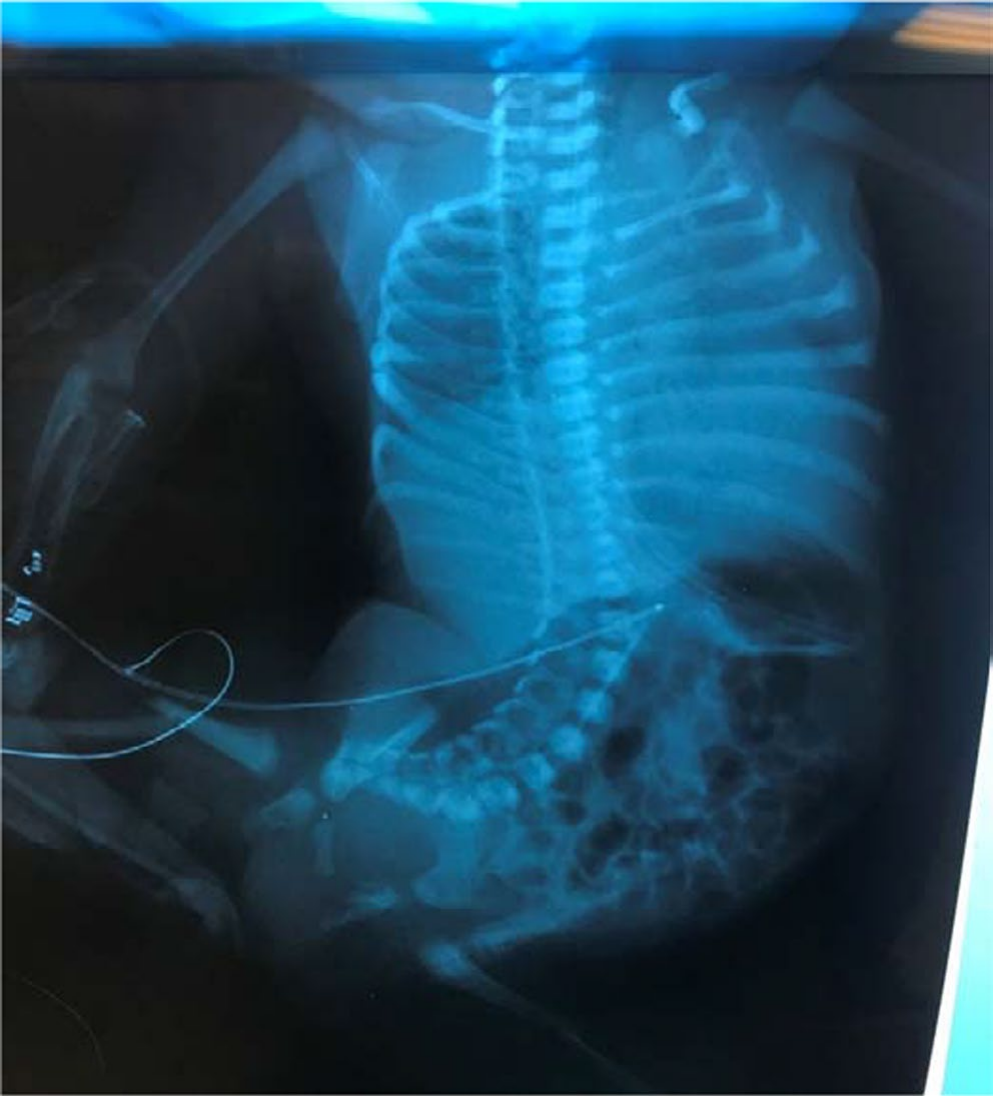
X‐rays soon after birth showing severe fixed scoliosis, pelvic obliquity, and bilateral femoral fractures

**Figure 2:**
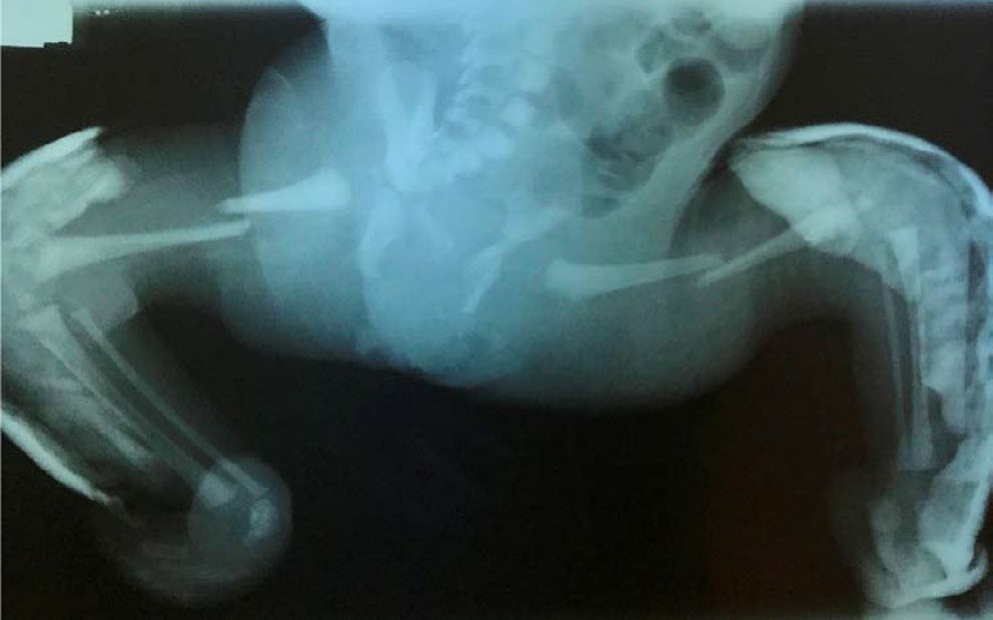
X‐ray showing bilateral femoral fractures. Casts were applied for fixed equinovarus feet

The baby was born by CS, because of breech position and abnormal prenatal US findings. The 1‐ and 5‐minute Apgar scores were 5 and 7, respectively. She was transferred to the neonatal intensive care unit for management of the multiple congenital deformities and mainly the peculiar shape of the body. The pelvis and lower legs were in vertical position with the spine. Both legs were clinically unstable, with crepitus. X‐ray examination revealed bilateral femoral fractures. Severe stiff scoliosis, affecting the lumbar spine, without vertebral congenital anomalies, was a remarkable finding (Figures 1 and 2). On examination, the upper limbs were in a stiff extension at elbows with <30 days flexion. Furthermore, the hands were stiff in mild flexion at the wrist and MP and IP joints, with absent skin creases. Pelvis was fixed on the right side of the body, with a mermaid‐like position of the neonate. The main feature was the lateral tilting of the lower spine and pelvis to the right side, resembling an L position of the spine and lower limbs. Both hips were stiff in 70 days of flexion. Knees were in fixed flexion position of 60 days, with only 30 days ROM. She had equinovarus stiff feet.

The infant had mild respiratory distress treated with oxygen for 19 days. US examination of heart, brain, liver, and kidneys was normal.

**Figure 3:**
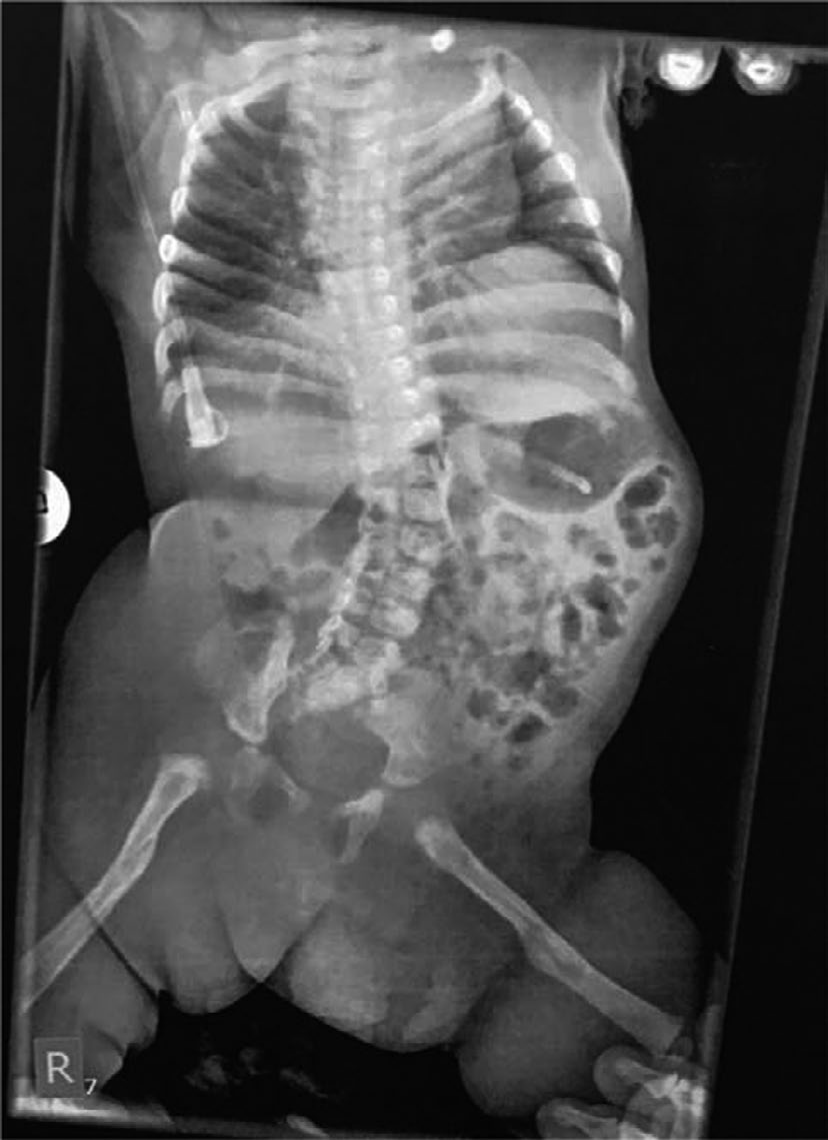
X‐ray showing complete healing of femoral fractures, improvement of scoliosis, and pelvic obliquity

Gallow's traction was applied immediately for the fractured femurs. We shifted the body and the pelvis gradually in her bed, while in traction, on the left side, in order to improve the position of the body. Simultaneously, casts for the clubfeet were also applied while in Gallow's traction. Callus formation of the fractured femora appeared on the 10th day. Healing of the fractures was completed on the 3rd week of life. At that time, the traction was removed. There was a pressure sore of the left calcaneus from the cast we used for the clubfeet. On clinical examination, both hips were stiff in flexion and abduction, with no signs of hip dislocation. Radiological examination confirmed that femurs healed nicely, while hips were in appropriate position. Considerable improvement of the spine and pelvis deviation was achieved (Figure 3).

**Figure 4:**
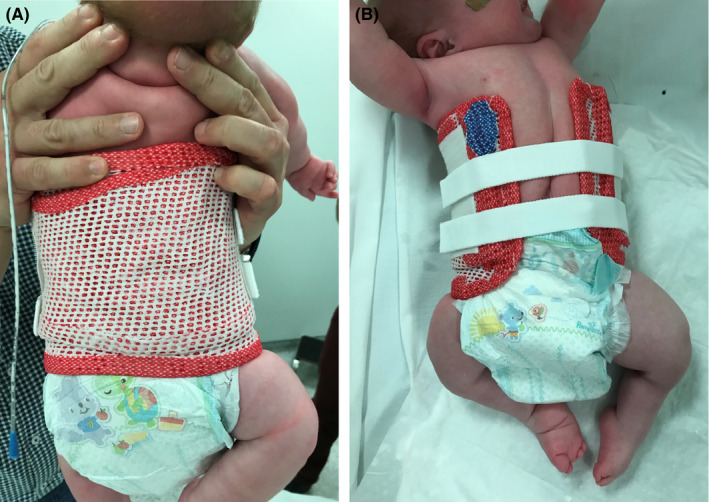
A and B, Cast for the correction of the spine scoliosis and pelvic obliquity posterior and anterior view

Treatment was focused on the spine, the stiff clubfeet, and the general management of an baby with severe contractures and malformation. Casts for the spine were used, in order to improve the lateral deviation of the spine and pelvis. The size of the cast was gradually increased at 20‐day interval (Figure 4A, B).

Currently, the clubfeet are treated with casts, while an achilles tenotomy is planned, before proceeding to final correction. In addition, gentle physiotherapy is applied in order to improve the position of the limbs.

## 3 DISCUSSION

Neonatal fractures occur in normal children usually as the result of maneuvers during difficult labor. Fractures of long bones are reported mostly affecting the femur and less commonly affecting the humerus.

Neonatal femoral fractures occur in cases of difficult breech delivery. Low or heavy birth weight, the advanced age of the mother, and uterine fibrinomas are predisposing factors.^7^ Neonatal femoral fracture is most common reported during a cesarean section. The small incision, the low position, and inappropriate movements in order to bring out the neonate to breath attribute to fractures. The technique used is also important. Adequate analgesia, sufficient incision, and smooth extraction of the neonate are proposed to avoid injuries.^2^

Bilateral femoral fractures are extremely rare in healthy neonates born by CS. Only one case of bilateral femoral fractures in a neonate delivered by CS has been published.^3^ Bilateral humerus fractures have been published in two case reports, both were CS, while the second one was in a twin pregnancy, with a right femoral fracture as well.^8^, ^9^

The presence of underlying disease as osteogenesis imperfecta, severe bone dysplasias, spinal muscular atrophy, or arthrogryposis increases the risk of perinatal femoral fractures.^4^, ^5^, ^6^ Fetal limb immobilization in malformation complex may contribute to the long bone propensity to fractures. A study investigated the incidence of femoral fractures in neonates with congenital arthrogryposis. In this study, conducted on 36 patients with arthrogryposis, 9 patients sustained 16 perinatal fractures. Femoral fractures were more common than humeral or tibial fractures.^5^

It has been proposed that cases with reduced fetal movements should be delivered with CS in a tertiary care center, in order to avoid perinatal fractures and provide possible pulmonary support.^10^ In our patient, the shape of the body and the stiffness were the main obstacle for the delivery during CS that was complicated with bilateral femoral fracture.

Another case of a new born with multiple fractures, affecting both femurs, was reported, that died at 21 days because of multiple organ failure from hypoxia and severe respiratory distress.^11^

Diagnosis of the fractures is usually based on clinical examination. The main features are swelling and reduced mobility of the fractured limb and severe discomfort in passive movements. Occasionally, fractures may be found incidentally during X‐ray investigation for other reasons, as respiratory distress.^12^ In the current case, femoral fractures were revealed immediately from the presence of instability and crepitus of the femurs. X‐ray examination confirmed the malformation complex with severe contractures, bone deformities, and presence of bilateral femoral fractures.

The current neonate had a unique stiff L shape of the body. That was due to the severe pelvic obliquity, with a tethering type of contracture on the right side. In the context of severe fixed deformity, we started treatment applying a cast that was changed every 2‐3 weeks, depending on the improvement of scoliosis.

There are several methods available for treatment of neonatal femoral fractures. In our case, we used Gallow's traction, as this method facilitated the appropriate handling of the neonate in the intensive care unit. Fractures united quickly, with excellent remodeling. Only a separation of the distal femoral epiphysis may require intervention, in case of instability and severe displacement. The use of Pavlik harness is considered easier to be applied in infants with femoral fractures, avoiding skin irritation.^13^, ^14^ However, the use of Pavlik harness was impossible in the current case because of the severe obliquity of the body.

In conclusion, neonates with malformation complex are at increased risk of femoral fractures. Early diagnosis and treatment are essential to improve skeletal deformities of the infant. Treatment with Gallow's traction is sufficient for the union of the fractures. Further treatment will follow for the stiff deformities of the limbs and spine.

## CONFLICT OF INTEREST

None of the authors has any conflict of interest.

## AUTHOR CONTRIBUTIONS

LN: is responsible for writing and editing the original draft. TK: is responsible for the investigation and collection of data. KP: is responsible for the data collection and assisting in editing. AE: is responsible for collection of data and review. CC: is responsible for investigation and assisting in editing.

## ETHICAL APPROVAL

Full consent has been given by the parents for publication of the case report.
